# Performance comparison of two microarray platforms to assess differential gene expression in human monocyte and macrophage cells

**DOI:** 10.1186/1471-2164-9-302

**Published:** 2008-06-25

**Authors:** Seraya Maouche, Odette Poirier, Tiphaine Godefroy, Robert Olaso, Ivo Gut, Jean-Phillipe Collet, Gilles Montalescot, François Cambien

**Affiliations:** 1 INSERM UMR S525, Faculté de Médecine Pierre et Marie Curie, Université Paris VI, 91 Boulevard de l'Hôpital, Paris 75634 Cedex 13, France; 2 Centre National de Génotypage, Evry, France; 3 INSERM U856, Institut de Cardiologie, Groupe Hospitalier Pitié-Salpêtrière, Paris, France

## Abstract

**Background:**

In this study we assessed the respective ability of Affymetrix and Illumina microarray methodologies to answer a relevant biological question, namely the change in gene expression between resting monocytes and macrophages derived from these monocytes. Five RNA samples for each type of cell were hybridized to the two platforms in parallel. In addition, a reference list of differentially expressed genes (DEG) was generated from a larger number of hybridizations (mRNA from 86 individuals) using the RNG/MRC two-color platform.

**Results:**

Our results show an important overlap of the Illumina and Affymetrix DEG lists. In addition, more than 70% of the genes in these lists were also present in the reference list. Overall the two platforms had very similar performance in terms of biological significance, evaluated by the presence in the DEG lists of an excess of genes belonging to Gene Ontology (GO) categories relevant for the biology of monocytes and macrophages. Our results support the conclusion of the MicroArray Quality Control (MAQC) project that the criteria used to constitute the DEG lists strongly influence the degree of concordance among platforms. However the importance of prioritizing genes by magnitude of effect (fold change) rather than statistical significance (p-value) to enhance cross-platform reproducibility recommended by the MAQC authors was not supported by our data.

**Conclusion:**

Functional analysis based on GO enrichment demonstrates that the 2 compared technologies delivered very similar results and identified most of the relevant GO categories enriched in the reference list.

## Background

Microarray-based gene expression analysis is a major component of functional genomics research. Using this approach, researchers can investigate the level of expression of all genes in a tissue or cell type in a single experiment [1]. Several questions and concerns about the reliability, reproducibility and quality of microarray data have been raised [2] and despite important recent advances in the evaluation of the existing technologies, some questions remain unanswered [3] and the scientist is often lacking arguments to decide on which approach is best suited for his purpose.

The recently published MicroArray Quality Control (MAQC) reports [4-9] provide rich information regarding intra- and inter-platforms reliability. The primary goal of the MAQC project was to evaluate the technical variability of DNA microarray results obtained with a number of different microarray technologies. The MAQC results showed relatively low technical variability in the intra-site and inter-site measurements, and high inter-platforms concordance for the thousands of genes identified as differentially expressed between 2 reference RNA samples explored under 4 titration conditions [10,11]. The project was focused on technical variability and was not trying to answer a biological question [12]. The present study was designed to evaluate the ability of 2 commonly utilized microarray technologies to answer a relevant biological question, namely the change in gene expression between resting monocytes and macrophages derived from these monocytes. M-CSF induced activation of monocytes for 6 days leads to differential regulation of a large number of genes, and offers the possibility to compare the microarray platforms across a wide range of differential gene expressions.

Lists of differentially expressed genes (DEG) between monocytes and macrophages were established according to various criteria and compared to a reference list derived from a large number of experiments using a third technology. Reproducibility and between platforms comparability was assessed on the whole content of each array and on a subset of well-matched transcripts common to the three investigated platforms. The biological relevance of the DEG lists was assessed by testing their enrichment in Gene Ontology (GO) [13] classes.

## Results

### Within and between platform consistency of expression data

**Figure 1:**
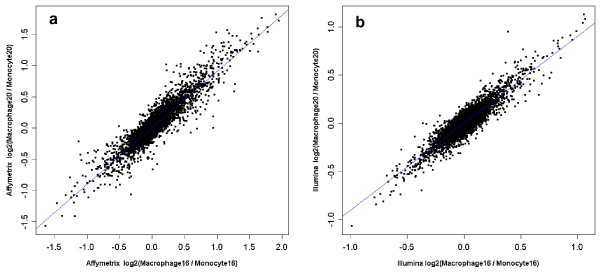
**Intra-platform reproducibility of the relative expression intensities**. Scatter plot comparison of the relative expression values (log 2 ratio of gene expression between macrophage and monocyte samples) of two different samples on Affymetrix (a) and Illumina (b) platforms. The blue line on each plot represents a regression line that best fits the plotted set of points. Both array types provide high inter-replicates reproducibility of the relative gene expression intensities.

For each platform, reproducibility of absolute and relative gene expression intensities between pairs of biological replicates within each sample type were examined on the subset of transcripts common to the three platforms. In monocyte samples, the inter-replicates correlation coefficients of absolute intensities ranged from 0.96 to 0.98 for Affymetrix, and from 0.98 to 0.99 for Illumina. In macrophages the respective ranges were 0.94 to 0.98 and 0.95 to 0.99. The correlations of relative expression (log ratio of hybridization signals between monocytes and macrophages mRNA) between pairs of replicates ranged from 0.82 to 0.94 for Affymetrix arrays and from 0.83 to 0.93 for Illumina arrays. Typical plots of relative expression intensities for a pair of samples are shown in Figure 1. These results suggest that both platforms deliver highly replicable signals. Additional file 1 shows the correlations coefficients of absolute and relative expression intensities between all pairs of replicates.

### Comparison of detection calls

For the subset of 19,404 transcripts (14,709 genes) common to the 2 compared platforms and the reference, the number of probes detected on each platform for each sample type is shown in Additional file 2 [see Additional file 3]. Concordance of detection calls between Affymetrix and Illumina was > 70%. Fifty eight Affymetrix probe sets called present in the 10 samples were not detected on the Illumina array (detection score < 0.80) and 185 Illumina probes detected with a score = 1 were called "absent" on the Affymetrix platform [see Additional file 3].

### Intra-platform reproducibility of replicate probes

Intra-platform reproducibility was also assessed by examining the expression levels of probes representing the same genes. Discordances were observed on the 2 platforms, the signal delivered by multiple probes tagging the same gene being uncorrelated and/or showing differences in expression levels in opposite direction. Examples of such discrepancies are available online [see Additional file 4].

### Differential expression analysis performed using all probes represented on each platform

The number of probes included in this analysis for each platform is provided online [see Additional file 5]. For a P-value corrected for multiple testing, Pc < 0.001, the analysis of Affymetrix data identified 4125 probe sets, corresponding to 2890 distinct genes differentially expressed between monocytes and macrophages. For the same level of statistical significance, the analysis of Illumina data identified 2841 differentially expressed probes, corresponding to 2399 unique genes. The reference list established using the RNG/MRC data with a Pc < 0.001 threshold included 8317 genes (the higher number of genes identified is explained by the larger sample size).

**Figure 2:**
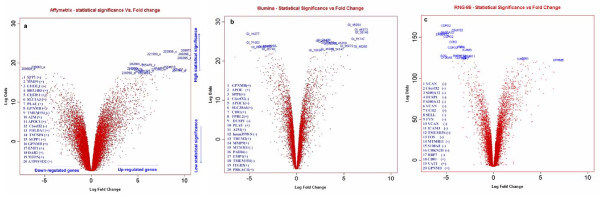
**Volcano plots representing the relationship between fold change and statistical significance**. On the x-axis are represented the log 2 fold change between the two groups (macrophages and monocytes). The vertical axis represents the log-Odds (B-Statistic) computed in Limma. Each gene is represented by a point and an up- and down-regulated gene appears symmetric. B statistics represents the log-odds that the gene is differentially expressed between the two groups. A low B-value indicates little evidence of differential expression. Highlighted genes represent the top 20 significant genes identified on Affymetrix (a), Illumina (b) and RNG-86 (c) platforms.

The 'Volcano plots' in Figure 2 provide a simultaneous representation of log2 fold change and statistical significance (log-odds) for the gene expression data obtained on the 2 compared and the reference platforms. For similar high log-odds the corresponding fold changes is smaller for data generated on the Illumina (the volcano plot is high and narrow) than on the Affymetrix platform (the volcano plot is high and large).

### Analysis based on a subset of well-matched transcripts common to the 3 platforms

The differential expression analysis was repeated using the subset of transcripts that were well matched across platforms (The number of probes included in this analysis is available online [see Additional file 5]). Table 1 provides the number of genes identified as differentially expressed on each platform using three different selection criteria: 1. a Pc < 0.001, 2. a Pc < 0.05 combined with a fold change > 2, and 3. the Best-3800 probes identified on each platform (as defined below).

**Table 1:** Results of the differential expression analysis

**Microarray Platform**	**Pc < 0.001**^#^	**Pc < 0.05 & FC > 2 **^##^	**Best 3800 probes**^###^
Affymetrix	**1877 genes** (1908 probe sets)	**1914 genes** (1945 probe sets)	**3738 genes** 0.05 < Pc < 10^-11^ 0.48 < |log 2 FC| < 10.38
Illumina	**1993 genes** (2010 probes)	**845 genes** (854 probes)	**3761 genes** 0.05 < Pc < 10^-11^ 0.26 < |log 2 FC| < 6.92
Reference list (RNG-86)	**5204 genes** (5677 probes)	**894 genes** (933 probes)	**3552 gene** 0.05 < Pc < 10^-79^ 0.34 < |log 2 FC| < 6.91

Number of differentially expressed genes between macrophage and monocyte samples using different criteria for selecting lists. The analysis was performed on the subset of well-matched transcripts common to the three platforms [see Additional file 5] for the number of probes included in this analysis for each platform).

# adjusted P-value (Pc) of the moderated t test.

## adjusted P-value of the moderated t test combined to fold change (FC).

### best overlapping lists of genes among platforms (see results section for details).

Based on the subset of well-matched transcripts and using the Pc < 0.001 criterion, the Affymetrix microarray identified 1877 genes (1908 probe sets) and the Illumina microarray identified 1993 genes (2010 probe IDs) while the reference list comprised 5204 genes corresponding to 5677 probes. The estimated FDR was < 1% on the three platforms [see Additional file 6]. The list of these genes and their associated statistics is provided as Additional data [see Additional file 7].

### Overlap in gene lists

**Figure 3:**
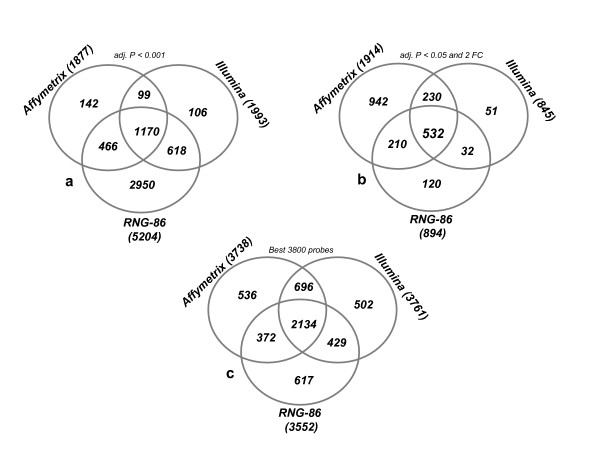
**Inter-platforms agreement in gene lists**. Venn diagrams showing the overlap of genes identified as differentially expressed between macrophage and monocyte samples in the 2 compared platforms and the reference. Three different criteria were used to select gene lists: (a) Pc < 0.001, (b) Pc < 0.05 combined to fold-change >2 as suggested in the MAQC project and (c) "Best-3800" probes on each platform. Only a set of transcripts common to the 3 platforms was used in this comparison. Gene lists include both up- or down- regulated genes.

**Figure 4:**
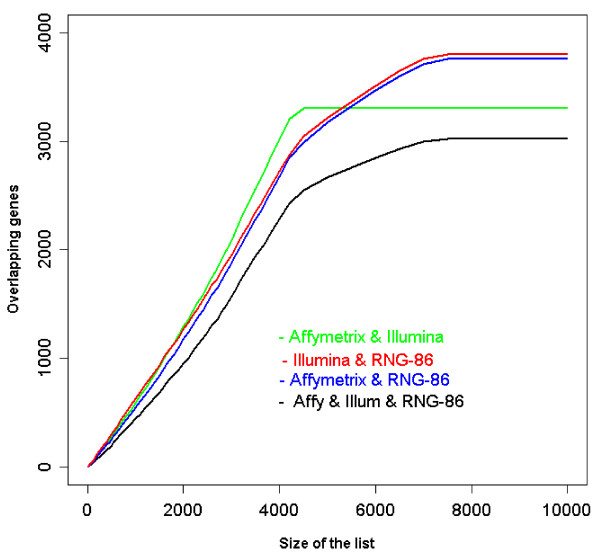
**Effect of gene list selection criteria on the degree of inter-platforms concordance**. Number of overlapping genes (y-axis) in the 2 and 3 lists of DEG according to the size of the list (x-axis). For each platform, the list was constituted by selecting DEG (Pc < 0.05), then within this list genes were ranked according to decreasing fold change. The number of overlapping genes between lists was calculated for increasing list size. When the number of probes in the lists was approximately 3800, the number of overlapping genes reached a plateau. The "best 3800" set of probes was defined accordingly.

**Figure 5:**
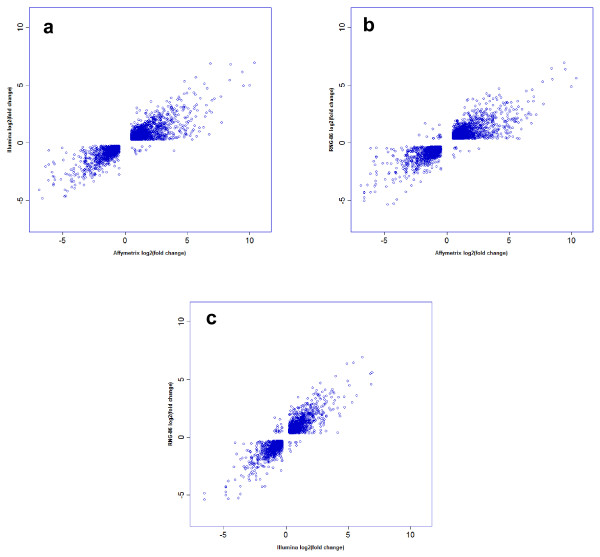
**Correlation of fold changes**. For each pair-wise comparison, Pearson's correlation coefficients of fold change were calculated and the direction of change was examined; Genes present in the top-left and bottom right quarters of each plots show changes in opposite direction. These genes are expected to overlap by chance.

The overlap among gene lists for the criteria used in Table 1 is reported in Figure 3(a). The Affymetrix and Illumina microarrays identified in common 1269 DEG, representing respectively ~68% and ~64% of all genes identified on each platform. Eighty seven percent and 89% of the genes in the Affymetrix and Illumina DEG lists respectively were also present in the reference list. When the lists were defined by a combination of a Pc < 0.05 and a fold change > 2, the number of genes identified in common by the three microarray platforms decreased to 532, the number of genes was still large in the Affymetrix list but was considerably reduced in the Illumina and reference lists. The analysis carried out using all probes represented on each platform yielded similar conclusion [Additional file 8]. The subset of "Best-3800" probes was established using the following procedure. 1. For each platform, the list of probes whose expression differed between monocytes and macrophages at a Pc < 0.05 was ranked by decreasing fold change. 2. For a given size of the list, the lists were compared and the number of overlapping genes (present in 2 or 3 lists) was plotted against the pre-specified size of the list (Fig. 4). As illustrated on the Figure 4 the number of overlapping genes increased with the size of the list and reached a plateau when the ~3800 top-ranked probes were selected in each list. Using the "Best-3800" probes, 2134 distinct genes were co-present on the 3 types of arrays (Fig. 3c). The correlation of fold changes between the ranked DEG lists was > 0.89 for the 2830 genes co-present on the Affymetrix and Illumina lists, > 0.88 for the 2506 genes co-present on the Affymetrix and RNG lists, and > 0.91 for the 2563 genes co-present on the Illumina and RNG lists. In addition, as the direction of change of expression of genes present by chance in the lists should frequently be discordant, the number of genes exhibiting discordant change for each pair of list was examined. As shown in Figure 5, discordant changes (genes present in the top-left and bottom-right quarters of each plot) were relatively few, representing 0.4%, 0.96% and 1.1% of the Affymetrix-Illumina, Affymetrix-reference and Illumina-reference list pairs, respectively.

### Gene Ontology (GO) comparison

Additional Table 4 online [see Additional file 9] shows, for each platform and using two gene list selection criteria, all GO categories over-represented (Pc < 0.05) in at least one of the three lists. Using the lists of "Best-3800" probes, sixteen GO biological processes were significantly enriched in at least one list; ten, 7 and 15 of these processes were enriched in the Affymetrix, Illumina and RNG-86 lists, respectively. The results of the GO analysis are summarized in Table 2 for the most relevant GO categories. For each gene list, the number of genes belonging to a particular GO category and the corresponding adjusted P-value are provided. For example, the "Immunity and defense" biological process category comprises 1,154, 1,124 and 1,123 genes among the 18,373, 16,592 and 17,550 genes represented on the Affymetrix, Illumina, and RNG arrays, respectively; these genes are highly over-represented in the "Best-3800" reference list (RNG-86) in which 326 out of 3,549 genes are mapped to this class [Table 4a, Additional file 9]. This over-representation is also observed for the Illumina and Affymetrix DEG lists which identified respectively 351 and 318 genes known to play a role in the "Immunity and defense" biological process. Using the Pc < 0.001 criterion [Table 4b, Additional file 9], the smaller number of genes within the DEG lists derived from the Affymetrix and Illumina platforms was reflected by a smaller number of genes present within relevant GO categories.

**Table 2:** Gene Ontology comparison.

**GO classes**	**Reference lists**			**Affymetrix lists**		**Illumina lists**		**RNG-86 lists**	
				
	Affy 18373	Illum 16592	RNG 17550	Best 3800 (n = 3735)	P < 0.001 (n = 1876)	Best 3800 (n = 3756)	Pc < 0.001 (n = 1990)	Best 3800 (n = 3549)	Pc < 0.001 (n = 5198)
Immunity and defense	1154	1124	1123	318 * (P < 10^-5^) ^§^	189 (P < 10^-8^)	351 (P < 10^-7^)	205 (P < 10^-7^)	326 (P < 10^-8^)	383 (P < 0.09)
Intracellular signaling cascade	818	779	802	217 (P < 0.05)	118 (P < 0.05)	214 (ns)	118 (ns)	208 (P < 0.05)	272 (ns)
Lipid, fatty acid and steroid metabolism	673	647	655	189 (P < 10^-3^)	107 (P < 10^-3^)	196 (P < 0.05)	119 (P < 10^-3^)	178 (P < 0.05)	223 (ns)
Apoptosis	470	457	458	130 (P < 0.05)	77 (P < 0.05)	127 (ns)	75 (ns)	132 (P < 0.05)	163 (ns)
Carbohydrate metabolism	520	502	496	132 (ns)	69 (ns)	147 (P < 0.05)	75 (ns)	143 (P < 10^-3^)	190 (P < 0.05)
Protein metabolism and modification	2426	2316	2340	584 (P < 10^-3^)	283 (ns)	624 (P < 10^-4^)	342 (P < 10^-3^)	600 (P < 10^-8^)	836 (P < 10^-6^)
Signal transduction	2973	2866	2979	617 (ns)	374 (P < 10^-3^)	591 (ns)	350 (ns)	565 (ns)	733 (P < 10^-6^)

GO biological process categories over represented in the lists of differentially expressed genes generated on each platform and according to two different selection criteria (the "Best 3800" probes or a Pc < 0.001). The table is ordered by the adjusted P-value of the test of association between RNG-86 list and GO categories. For each platform, list of DEG was compared to the list of all genes represented on the array. Genes belonging to several GO classes are included several times, once for each GO class that is associated with this gene.

* Number of genes in the Affymetrix list annotated to the GO biological process Immunity and defense.

§Adjusted P-value derived from the binomial statistics testing the significance of the enrichment of the Immunity and defense GO category in Affymetrix list.

When the comparison of the two platforms was focused on a common set of genes represented on the 3 array types, the Illumina list tended to include a larger number of genes belonging to the relevant GO categories than the Affymetrix list. This observation was made for both criteria used to define gene lists. Conversely, the results of the GO analysis based on the whole content of each platform revealed that the Affymetrix platform identified a larger number of genes within GO categories over-represented in the reference list [Table 4c, Additional file 9].

Given the high similarities between the Affymetrix and Illumina platforms in term of number of genes identified and P-values it was important to check whether these similarities reflected the identification of the same genes by both platforms. Table 3 reports the number of overlapping genes in the "Best-3800" DEG lists according to GO categories. The last column in the table shows, for each GO category, the number of genes present in at least one of the lists. For example, within the "Immunity and Defense category", 427 genes were present in at least one of the lists; among them, 235 (55%) were present in the 3 DEG lists. The good performance of the 2 compared platforms relative to the reference is striking. In term of complementarity of platforms, if the genes identified by only one platform are excluded to limit the number of false positives, columns 4 and 5 provide the numbers of genes not identified by the Illumina and Affymetrix platforms respectively. These numbers are relatively small suggesting that in the context of this study, each of the 2 compared platforms provide rather complete information that is little complemented by the other platform.

**Table 3:** Degree of overlap in Gene Ontology categories over-represented in the lists of genes selected with the "Best-3800" probes criterion.

**GO Categories**	**Present in the 3 lists**	**Affy and Illum only**	**Affy and RNG-86 only**	**Illum and RNG-86 only**	**Affy only**	**Illum only**	**RNG-86 only**	**All**
Immunity and defense	235	45	21	32	17	39	38	427
Intracellular signaling cascade	137	35	14	23	31	19	34	293
Lipid, fatty acid and steroid metabolism	125	30	21	20	13	21	12	242
Apoptosis	86	18	11	14	15	9	21	174
Carbohydrate metabolism	92	20	11	18	9	17	22	189
Protein metabolism and modification	355	106	47	77	76	86	121	868
Signal transduction	393	97	55	47	72	54	70	788

## Discussion

Monocytes and macrophages are important players in the immediate response to foreign agents and in the development of the adaptive immune response [14]. Circulating monocytes are derived from specific myeloid progenitor cells and under particular conditions they may enter the arterial wall and mature into macrophages. Macrophages are involved in the initial process of atherosclerotic plaques formation, and they are also involved in the inflammatory events that trigger the rupture of atherosclerotic plaques and clinical events [15]. Investigating the biology of human blood monocytes, a relatively easily accessible cell, and macrophages is therefore of crucial interest for atherosclerosis research. The domain has been profoundly transformed by the recent availability of expression microarrays and we may be at the beginning of a new era in clinical and epidemiological research in which this technology will be used to investigate gene expression in circulating cells to predict occurrence, severity or evolution of disease as well as responses to treatment. Assessing the reproducibility and biological relevance of available microarray technologies is therefore of major importance.

The Illumina and Affymetrix microarray technologies differ in many aspects. While Affymetrix arrays use a set of different 25-mer probes synthesized *in situ *[16] to characterize gene expression, Illumina arrays utilize multiple copies of a single 50-mer probe attached to micro beads to quantify targets levels [17]. In addition, Affymetrix probes are located at pre-specified locations on the array while on the Illumina array, ~30 beads for each probe are randomly distributed on the array and decoded using specific tagging sequences. These and other technical differences may lead to different results; data are therefore needed to comparatively assess the technologies. Fortunately, recent large scale studies have provided a wealth of data generated by a number of array technologies including those investigated in the present study. According to the results of the MAQC project the Affymetrix and Illumina arrays provide highly reproducible results [Fig. 2 in [4]] that correlate well with single gene expression measurements obtained by RT-PCR and TaqMan assays [Fig. 4 in [7]]. Former comparisons of the Affymetrix and Illumina technologies also based on a dilution study design led to the conclusion that both arrays deliver highly correlated results, especially for relatively high expression levels [18].

Questions remain however regarding the biological relevance of the gene expression measurements and differential patterns of expression delivered by both approaches and their possible complementarity. In this paper we focused on the relative abilities of Affymetrix and Illumina microarrays to characterize the change in gene expression that parallels the maturation of human blood monocytes into macrophages. To allow this comparison, a reference panel of differentially expressed genes was identified using a large number of samples hybridized to a third type of microarray (2-color RNG/MRC microarrays [19]. It is well established that macrophages exhibit an important heterogeneity which depends in vitro on the type and duration of the stimulus used to generate them from monocytes and in vivo on their cellular and molecular micro-environment [20]. In addition, the response of macrophages to stimuli is dynamic and profound, implying important temporal changes of gene expression that parallel functional changes. The variability of expression of this cellular model may therefore be difficult to control even in rigorously designed experiments, in which the various sources of variability from the first stages of blood processing to the preparation of RNA samples are controlled. Given these premises it was relatively reassuring to note the high inter-replicates correlation coefficients of the absolute and relative intensities delivered by both platforms which most of the times exceeded 0.90 in this study. The experimental protocol generated a large number of differentially expressed genes. Using all probes present on each platform and the Pc < 0.001 criterion, the Affymetrix and Illumina lists included 2890 and 2399 genes respectively, while 8317 unique genes were present in the reference list. In our analysis, the two-color microarray experiment is not considered as a "gold-standard" but as a reference used to compare the two commercial arrays. The much larger number of hybridizations conducted on the RNG/MRC platform led to theidentification of a large number of differentially expressed genes, many of which were enriched in pathways relevant to monocyte and macrophage biology. Using a similar monocytes/macrophage model other authors [21] observed that 4130 of 13582 genes were differentially expressed. Such massive change may not reflect the common situation where researchers investigate highly specific modifications of expression levels. But changes affecting a wide range of differential expression levels are of interest to compare microarray technologies as subtle modifications of expression generated by a specific differentiating factor may be better detected by one type of array while another type of array may be more suitable to evaluate modifications generated by another differentiating factor.

Several papers have shown that the methods used to pre-process and analyze gene expression data can strongly influence the results [6,22,23]. In the present study, we found that pre-processing methods (background correction and normalization) can have a profound influence on the degree of agreement in gene lists among the platforms. For the data generated on the RNG/MRC platform we investigated several background correction and within/between array normalization methods implemented in the Limma and marray packages. We found that the use of different normalization and background adjustment methods can have a profound influence on the number of genes found to be differentially expressed on this platform (data not shown). To limit this problem, we applied equivalent normalization and statistical analysis methods to generate the DEG lists for the 2 compared platforms and the reference.

Discrepancies of results between probes representing the same gene, observed on the Affymetrix and Illumina platforms (See results) can be attributed to several factors, including cross-hybridization problems, probe binding to alternatively spliced transcripts and technical noise.

The most important factor influencing the discordance among microarray platforms is the criterion used to define the lists of differentially expressed genes [24,25]. To define gene lists we used the Pc-value, fold change and the DEG list size. The three different criteria are, of course, not independent but their combination may lead to very different results. One of the conclusions of the MAQC project was that previously reported lack of agreement among lists of genes generated on different platforms was due to the use of a statistical significance threshold to define DEG lists. MAQC's authors [4,6,10] and others [26] recommended selecting gene lists with a non-stringent P-value cutoff combined to fold-change. Our results (Table 1, 2^nd ^column) show that when a non stringent Pc-value (< 0.05) combined to a fold change >2 are used, the number of differentially expressed genes is considerably larger for the Affymetrix than for the Illumina platform. The reason for this discrepancy is evident when examining the Volcano plots (Fig. 2) which show that for similar statistical significance, the fold-change is much less important for the Illumina than for the Affymetrix data. A fixed fold change is therefore inappropriate for comparing these 2 platforms. On the other hand, a relatively more stringent Pc < 0.001 criterion, irrespective of fold change, provided more consistent results between the 2 platforms (Fig. 3). Using this criterion, ~90% of the 1993 genes in the Illumina list and ~87% of the 1877 in the Affymetrix list were also present in the reference list. We then used a selection criterion combining a non stringent statistical significance threshold (Pc < 0.05) and a "relative" fold change. In order to take into account the inter-platforms differences in the magnitude of change, the DEG list was defined not by a fold change threshold but according to the size of the list (the same for each platform). As shown in Figure 4, using this criterion, the overlap among the lists of the different platforms increases with the size of the list but reaches a plateau on the Affymetrix and Illumina lists when the size of the lists reaches approximately 3800. The list of "best 3800" was therefore considered as the most parsimonious list maximizing the overlap among platforms. As expected, the "best 3800" list corresponded to different fold change thresholds according to the platforms: 1.40 for Affymetrix, 1.21 for Illumina and 1.27 for the reference.

A major aspect of microarray data analysis is the focus on classes of functionally related genes rather than on single genes [27]. This approach has not only increased the relevance of experiments conducted on a small number of biological replicates but most importantly it offers new perspectives that are only beginning to be explored in the area of systems biology. Data analysis approaches investigating classes of related genes necessitate 2 steps: the selection of gene lists and the test of an over/under representation of genes belonging to these lists in a priori defined sets of genes representing functional classes. Because the main interest is in statistical testing at the level of the functional classes, it may be appropriate to use a non-stringent selection criterion to constitute gene lists.

The approach used in this report to characterize gene enrichment based on GO classes is one among several possible approaches that rely on various ontology or pathway databases and utilize different statistical methods to define gene lists and enrichment [28].

Using the Pc < 0.001 criterion, a smaller number of DEG were identified within each GO category than when using the "Best-3800" gene list. This reduced number of identified genes was not always associated with a proportional decrease of the corresponding P-values for GO enrichment. This may reflect both a greater specificity of the gene lists established using the Pc < 0.001 criterion compared to the "Best-3800" genes criterion and a weaker influence of the adjustment for multiple testing.

The most relevant GO categories identified by the reference list (RNG-86) were also significantly over-represented in the lists identified by the Affymetrix and Illumina platforms. They included several expected categories, such as "Immunity and defense", "Apoptosis", "Intracellular signalling cascade" as well as categories characterizing general cellular metabolic activities, which are considered as prominent features of macrophages differentiation [29], such as "Protein metabolism", "Carbohydrate metabolism" and "lipid, fatty acid and steroid metabolism". The complete lists of GO categories over-represented in the lists of genes differentially expressed between monocytes and macrophages are reported in additional Table 4 online [see Additional file 9].

Overall, the Illumina platform performed slightly better than the Affymetrix one when the platforms were compared using the genes present on both types of array; whereas the reverse was true when the comparison was based on the whole set of gene present on each platform. This is certainly explained by the larger number of genes represented on the Affymetrix than the Illumina array (20,252 and 16,756 respectively, see methods). Despite these differences, the results of the GO analyses were remarkably similar between platforms in term of identified GO categories, number of genes present within identified GO categories and statistical significance.

## Conclusion

In conclusion, this work compared the ability of two commonly used microarray platforms to characterize the differential gene expression profiles of human blood monocytes and macrophages. A third microarray technology applied to a larger number of experiments was used as reference. The results show that the criterion used to select the gene lists may considerably affect the results. A selection procedure coupling a non stringent P-value and "relative" fold-change identified a list of approximately 3800 probes that optimized the overlap among DEG lists identified on each platform. A functional analysis based on GO enrichment demonstrated that the 2 compared technologies delivered very similar results and despite the small number of samples, they identified most of the relevant GO categories enriched in the reference list.

## Methods

### RNA samples

86 samples were obtained from patients with symptoms of acute coronary syndrome who had undergone coronary angiography at the department of cardiology of the Pitié-Salpêtrière Hospital, Paris and who had one stenosis > 50% diagnosed in at least one major coronary artery. This study was approved by the ethic committee of Pitié-Salpêtrière Hospital and informed consent was obtained from all participants.

### Experimental design

The objective was to compare 2 commercial microarrays, Illumina Bead Chip Human-6 V1 [17] and Affymetrix HGU133plus 2.0 [16] between each other and to a reference list of DEG established using the academic Réseau National des Génopoles/Medical Research Council (RNG/MRC) two-color chip [19]. Monocyte and monocyte-derived macrophage RNA samples from 5 individuals were hybridized to the 2 types of microarrays. To constitute the reference DEG list, 86 monocyte and 86 macrophage samples (38 pools of 2 samples and 10 individual samples for each type of RNA) were hybridized to RNG/MRC microarrays (RNG-86).

### mRNA preparation, amplification and hybridization on microrrays

See additional material online [see Additional file 10] for a detailed description of the protocols. In short, blood was drawn under standardized conditions in EDTA tubes: 40 ml samples were collected immediately after coronary angiography and stored at 4°C. Monocytes isolation was performed within 2 hours after blood drawing. After density gradient centrifugation and washing, PMBC were mixed with CD14 coated beads (MACS, Miltenyi Biotec). To induce phagocytic differentiation, a fraction of the monocytes was incubated for 6 days with macrophage colony stimulating factor (M-CSF, SIGMA). RNA extraction from monocytes and macrophages was done using RNAeasy minikit (Qiagen). All RNA preparations were checked with an Agilent Bioanalyser (RNA 6000 nano-kit) and only RNA with RNA integrity number (RIN) > 8 were accepted for RNA amplification. Amplification of RNA, hybridization, image processing, and raw data extraction were performed using protocols suitable for each platform.

### Background adjustment, normalization and probes filtering

For all platforms, low-level analysis (background adjustment, filtering, quality assessment, and normalization) was performed in the statistical environment R [30]. For Illumina data, quality control and pre-processing were performed using the Bioconductor packages BeadArray and BeadExplorer [31]. Bead-averaged data was normalized using a quantile normalization method [32]. For Affymetrix data, probe level data was summarized using the 'Affy' package; only perfect match (PM) probe intensities were background corrected (RMA method). After background adjustment, PM intensities were summarized (Median polish method) into one expression value for each probe set. The 10 arrays were normalized together using the quantile method. Data generated on the RNG/MRC platform were processed using the Bioconductor packages marray [33] and Limma [34]. Within array print-tip loess normalization on the background-corrected red and green intensities (normexp method) was performed for each spot followed by between array quantile normalization. The number of probes filtered out and included in the statistical analyses for each platform is available online [see Additional file 5]; in particular the number of probes included in the analysis based on a common list of transcripts after filtering on detection calls was 8310, 8319 and 9777 in the Illumina, Affymetrix and RNG/MRC lists, respectively (see Additional file 10 for a description of the filtering procedure).

### Gene lists annotation

Gene lists were annotated using the Bioconductor chip annotation packages (hgu133plus2, IlluminaHumanv1, and hs25kresogen for Affymetrix, Illumina, and RNG/MRC, respectively). Entrez gene IDs were used to compare gene lists generated on the three platforms. In addition, annotation data provided by chip manufacturers were used. The NetAffx Analysis Center [35] was used to map probe sets and annotation information. For the RNG/MRC array, further annotation information was retrieved from the Mediante database [36]. According to the Bioconductor annotation packages, Affymetrix, Illumina and RNG/MRC arrays are informative on 20252, 16756, 18339 Entrez IDs, respectively.

### Cross-platform probe matching and common list construction

Probe matching information from the MAQC project [37] was used to map probe sequences between Illumina and Affymetrix. In addition a mapping file comparing probe content of the Illumina Human-6 v1 array with that of the Affymetrix HGU133plus2.0 was provided by Illumina [38]. Probe mapping between the Affymetrix and the RNG/MRC arrays was available from the Mediante database [36] and allowed the mapping of RNG/MRC ids to the RefSeq mRNA human database [39] and to the probes IDs from the two other microarray platforms. This information was used to establish a common set of well-matched transcripts present on the three platforms. Probes called absent in all samples on the Affymetrix platform and probes with detection score < 0.80 in all samples on the Illumina platform were removed. When a gene was represented on a given platform by more than one probe, all probes were included in the list of common set of transcripts. The resulting list of 19,404 human transcripts (14,709 genes) was used in the inter-platforms comparison based on a common set of transcripts.

### Differential expression analysis

For all platforms, differential expression analysis was performed in the same way using the Linear Model for Microarray Data (Limma) [40]. Limma provides functions for fitting a linear model to the expression data for each gene and performing moderated t-tests which is an empirical Bayes modification of the t-test to improve variance estimation for small sample sizes. Results from Limma included log fold change, moderated t-test (t-like statistics), P-value and log odds. Benjamini and Hochberg correction method was used to account for multiple testing [41]. The false discovery rate (FDR) was estimated by calculating q-values as described in [42] based on the P values derived from the moderated t-test statistics. This estimation was performed using the Bioconductor LBE (Location Based Estimation) package [43]. For each platform, lists of DEG selected using different statistical significance and fold change thresholds were generated and annotated. At similar significance thresholds, the lists were compared across platforms and to the reference list.

### Gene Ontology analysis

To identify Gene Ontology (GO) classes significantly enriched in the gene lists generated for each platform, a functional analysis was performed using the Panther Protein Classification system [44]. For each platform, list of DEG were annotated with Entrez IDs, uploaded to the Panther system, mapped onto GO Biological process classes that were significantly represented, and statistically compared to the list of all genes represented on the array. The binomial test [45] was used to look for under- and over-represented GO categories and a modified Bonferroni correction for multiple comparisons was applied. Benferroni method was modified to account for the dependency between GO terms since all genes annotated to a given GO node are also annotated to all its parents.

## Abbreviations

DEG: Differentially Expressed Genes; RNG/MRC: Réseau National des Génopoles/Medical Research Council; GO: Gene Ontology; FDR: False Discovery Rate; Limma: Linear Model for Microarray data; MAQC: MicroArray Quality Control; M-CSF: Macrophage colony-stimulating factor; Pc: P-value corrected.

## Availability

Raw and processed data from the three microarray platforms have been deposited in the National Center for Biotechnology Information Gene Expression Omnibus (GEO, ) public repository, and they are accessible through GEO Series accession number GSE10213 (Illumina data), GSE11430 (Affymetrix data) and GSE10220 (RNG/MRC data).

All R scripts used in this analysis are available upon requests.

## Authors' contributions

SM performed the bioinformatic analysis and drafted the manuscript under the supervision of FC, OP constructed the DNA bank and conducted the two-color experiments with TG, RO and IG performed the Illumina and Affymetrix microarray experiments, J–PC and GM supervised the patients recruitment; FC conceived and coordinated the study and finalized the manuscript. All authors read and approved the final version of this manuscript.

## Supplementary Material

Additional file 1 **Within and between platforms consistency of expression data**. Pearson correlations coefficients of absolute and relative expression intensities between all pairs of replicates. Click here for file

Additional file 2 **Comparison of detection calls**. Intra- and inter-platforms concordance of detection calls Click here for file

Additional file 3 **Discordance of detection calls between Affymetrix and Illumina**. Discrepancies in detection calls between Affymetrix and Illumina. Click here for file

Additional file 4 **Intra-platform reproducibility of replicate probes**. Examples of discrepancies in gene expression levels measured by probes representing the same genes. Click here for file

Additional file 5 **Probes filtering**. For each platform, the number of probes filtered out and the number of probes included in each analysis. Click here for file

Additional file 6 **False Discovery Rate (FDR) estimation**. The plot shows the histogram of the p-values derived from the Limma moderated t statistics, q-values versus p-values and the expected proportion of FDR estimated on Affymetrix (a), Illumina (b) and RNG-86 (c) platforms. The proportion of true null hypotheses (∏0) was estimated to be 0.273, 0.259 and 0.355 on the Affymetrix, Illumina and RNG-86, respectively. Click here for file

Additional file 7 **List of genes identified as differentially expressed between monocytes and macrophages**. Genes identified as differentially expressed between monocyte and macrophage samples on the three platforms (adjusted P-value < 0.001). Click here for file

Additional file 8 **Degree of overlap in lists of differentially expressed among the three platforms**. Venn diagrams of the number of genes identified as differentially expressed between monocyte and macrophage samples. Analysis was performed on all probes represented on each platform Affymetrix: 54,613 probe sets, Illumina: 47,296 probe IDs, RNG/MRC: 25,951 (only control probes and bad spots were filtered out). Results are shown for adjusted P-value < 0.001 threshold (a) and adjusted P-value < 0.05 combined to fold change > 2 (b). Click here for file

Additional file 9 **Gene Ontology enrichment comparison**. GO enrichment comparison of the lists of differentially expressed genes selected using two criteria. Click here for file

Additional file 10 **Additional material**. mRNA preparation, amplification, hybridization to the 3 array types, image processing, raw data and probes filtering. Click here for file
