# Supplementary material for: Performance comparison of two microarray platforms to assess differential gene expression in human monocyte and macrophage cells
Source: BMC Genomics. 2008 Jun 25;9:302. doi: 10.1186/1471-2164-9-302 (PMC2464609; doi:10.1186/1471-2164-9-302)
Supplement: Additional file 1 — Within and between platforms consistency of expression data. Pearson correlations coefficients of absolute and relative expression intensities between all pairs of replicates. [file 1471-2164-9-302-S1.doc]

**Additional Table 1. Intra- and inter-platforms concordance of expression levels.** For each platform, Pearson correlation coefficients between each array pair within each sample type were calculated on the subset of well matched transcripts. Black and red represents correlation coefficients of absolute expression intensities calculated on Affymetrix and Illumina, respectively (A & B). The correlation coefficients of the relative intensities (log-ratio of macrophage and monocyte samples) are shown in (C). These results show that both Affymetrix and Illumina platforms had a high level of inter-replicates reproducibility.

A) Pearson’s correlation coefficients of absolute expression intensities in monocyte samples

|  | **mono16** | **mono20** | **mono21** | **mono26** | **mono28** |
| --- | --- | --- | --- | --- | --- |
| **mono16** | 1  1 | 0.97  0.98 | 0.97  0.98 | 0.98  0.98 | 0.97  0.98 |
| **mono20** |  | 1  1 | 0.97  0.98 | 0.98  0.98 | 0.96  0.98 |
| **mono21** |  |  | 1  1 | 0.98  0.98 | 0.97  0.98 |
| **mono26** |  |  |  | 1  1 | 0.97  0.99 |
| **mono28** |  |  |  |  | 1  1 |

B) Pearson’s correlation coefficients of absolute expression intensities in macrophage samples

|  | **macro16** | **macro20** | **macro21** | **macro26** | **macro28** |
| --- | --- | --- | --- | --- | --- |
| **macro16** | 1  1 | 0.95  0.98 | 0.96  0.97 | 0.96  0.98 | 0.96  0.98 |
| **macro20** |  | 1  1 | 0.97  0.97 | 0.98  0.98 | 0.94  0.96 |
| **macro21** |  |  | 1  1 | 0.98  0.98 | 0.95  0.96 |
| **macro26** |  |  |  | 1  1 | 0.94  0.95 |
| **macro28** |  |  |  |  | 1  1 |

|  | **Sample 16** | **Sample 20** | **Sample 21** | **Sample 26** | **Sample 28** |
| --- | --- | --- | --- | --- | --- |
| **Sample 16** | 1 1 | 0.92 0.90 | 0.93 0.90 | 0.92 0.89 | 0.89 0.89 |
| **Sample 20** |  | 1 1 | 0.92 0.88 | 0.94 0.93 | 0.82 0.83 |
| **Sample 21** |  |  | 1 1 | 0.94 0.90 | 0.86 0.85 |
| **Sample 26** |  |  |  | 1 1 | 0.83 0.83 |
| **Sample 28** |  |  |  |  | 1 1 |

C) Pearson’s correlation coefficients of relative expression intensities
