# Supplementary material for: Performance comparison of two microarray platforms to assess differential gene expression in human monocyte and macrophage cells
Source: BMC Genomics. 2008 Jun 25;9:302. doi: 10.1186/1471-2164-9-302 (PMC2464609; doi:10.1186/1471-2164-9-302)
Supplement: Additional file 2 — Comparison of detection calls. Intra- and inter-platforms concordance of detection calls [file 1471-2164-9-302-S2.doc]

**Additional Table 2. Intra- and inter-platforms concordance of detection call.** For a common subset of 19,404 transcripts (14,709 genes) corresponding to 15,278 Affymetrix probe sets and 15,285 Illumina probe IDs, the detection calls (i.e. a measure indicating whether a given target sequence is expressed above the background in a sample) on the two platforms was compared. For Illumina a detection score < 0.80 was used as a threshold of detection. All Affymetrix probe sets called present (“P” calls) in the 10 samples were considered as detected. The number of probe detect in each sample within each cell type is shown. Inter-platform concordance of detection call was > 70 %.

| **Number of probes detected in each sample** | | | | | | |
| --- | --- | --- | --- | --- | --- | --- |
| **a)** **Monocyte samples** | | | | | | |
| Platform | **mono16** | **mono20** | **mono21** | **mono26** | **mono28** | **Detected in all monocyte**  **samples** |
| Affymetrix  (15,278 probe sets) | 7219 | 6991 | 7152 | 7009 | 7202 | 6500 |
| Illumina (15,285 probes) | 9656 | 9991 | 9697 | 9580 | 9669 | 7680 |
| Detected on Affymetrix and Illumina arrays | 6711 | 6555 | 6661 | 6531 | 6638 | 5809 |
| **b) Macrophage samples** | | | | | | |
| Platform | **macro16** | **macro20** | **macro21** | **macro26** | **macro28** | **Detected in all macrophage**  **samples** |
| Affymetrix (15,278 probe sets) | 7761 | 7244 | 7482 | 7398 | 7528 | 6781 |
| Illumina (15,285 probes) | 10365 | 10310 | 10089 | 10252 | 9995 | 8158 |
| Detected on Affymetrix and Illumina arrays | 7280 | 6791 | 7482 | 6960 | 7014 | 6119 |
