# Supplementary material for: Performance comparison of two microarray platforms to assess differential gene expression in human monocyte and macrophage cells
Source: BMC Genomics. 2008 Jun 25;9:302. doi: 10.1186/1471-2164-9-302 (PMC2464609; doi:10.1186/1471-2164-9-302)
Supplement: Additional file 5 — Probes filtering. For each platform, the number of probes filtered out and the number of probes included in each analysis. [file 1471-2164-9-302-S5.doc]

**Table 3. Filtering results**: for each platform, the number of probes filtered out and the number of probes included in each analysis.

| Microarray  Platform | Number of features on the arrays | Number of probes included in the analysis based on common list of transcripts(after filtering on detection calls) | Number of probes included in analysis using all probes (only control probes and probes with bad flags are filtered out) | Number of control probes | Number of probes called absent in all samples | Number of probes in the common list |
| --- | --- | --- | --- | --- | --- | --- |
| Illumina | 48876 | 8 310 | 47 296 | 1580 | 11857 (~ 25.07) * | 15285 |
| Affymetrix | 54675 | 8 319 | 54 613 | 62 | 34565 (~ 63.2 %)** | 15287 |
| RNG/MRC | 26496 | 9 777 | 25 951 | 545 | NC | 17480 |

* Detection score < 0.80 was used for the Illumina data (detection score close to 1 indicates a high confidence of detection.
** probe sets with “A” calls in all samples.
