# Supplementary figures and images for: Performance comparison of two microarray platforms to assess differential gene expression in human monocyte and macrophage cells
Source: BMC Genomics. 2008 Jun 25;9:302. doi: 10.1186/1471-2164-9-302 (PMC2464609; doi:10.1186/1471-2164-9-302)

## Slide 1
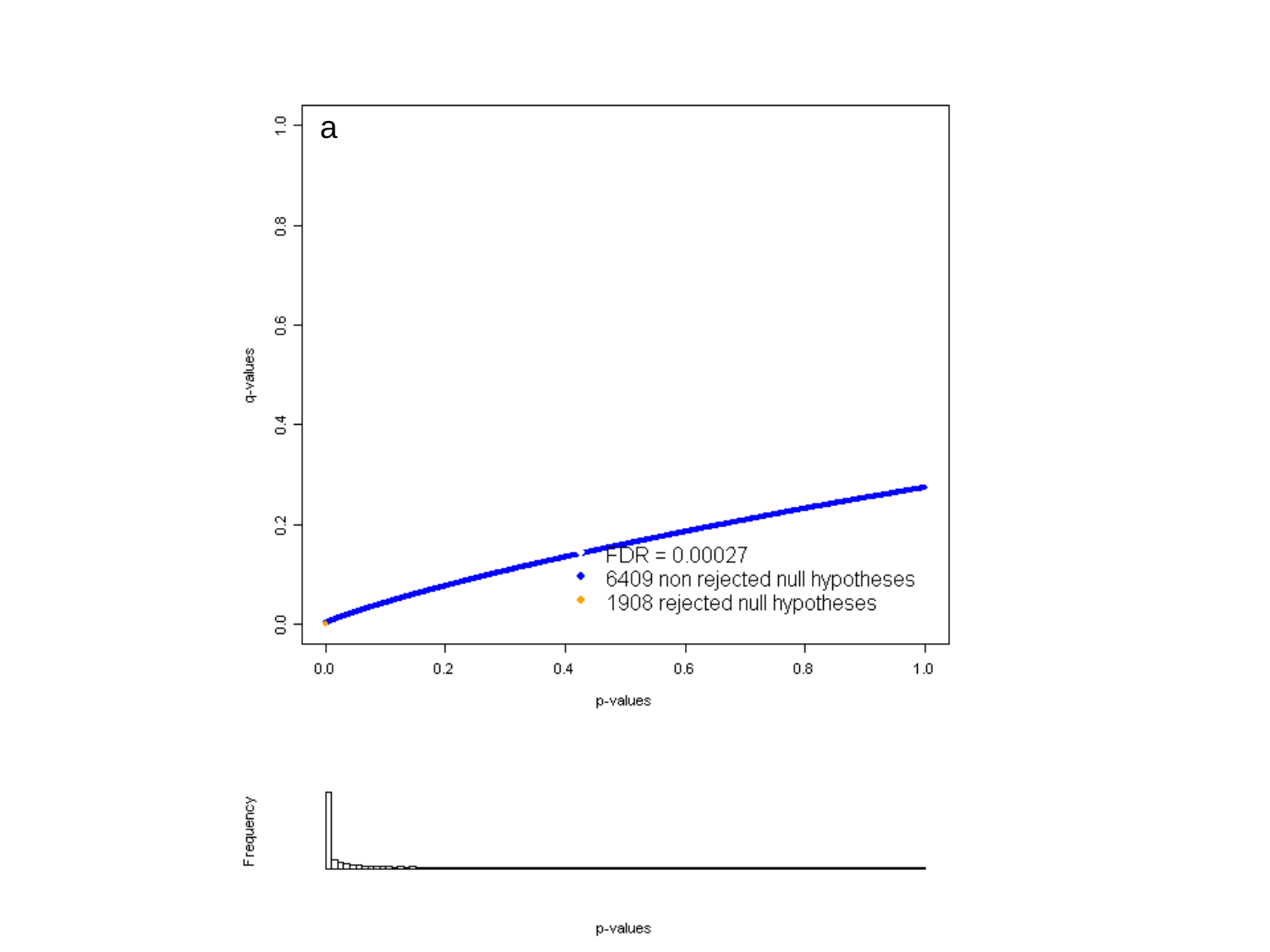

a

## Slide 2
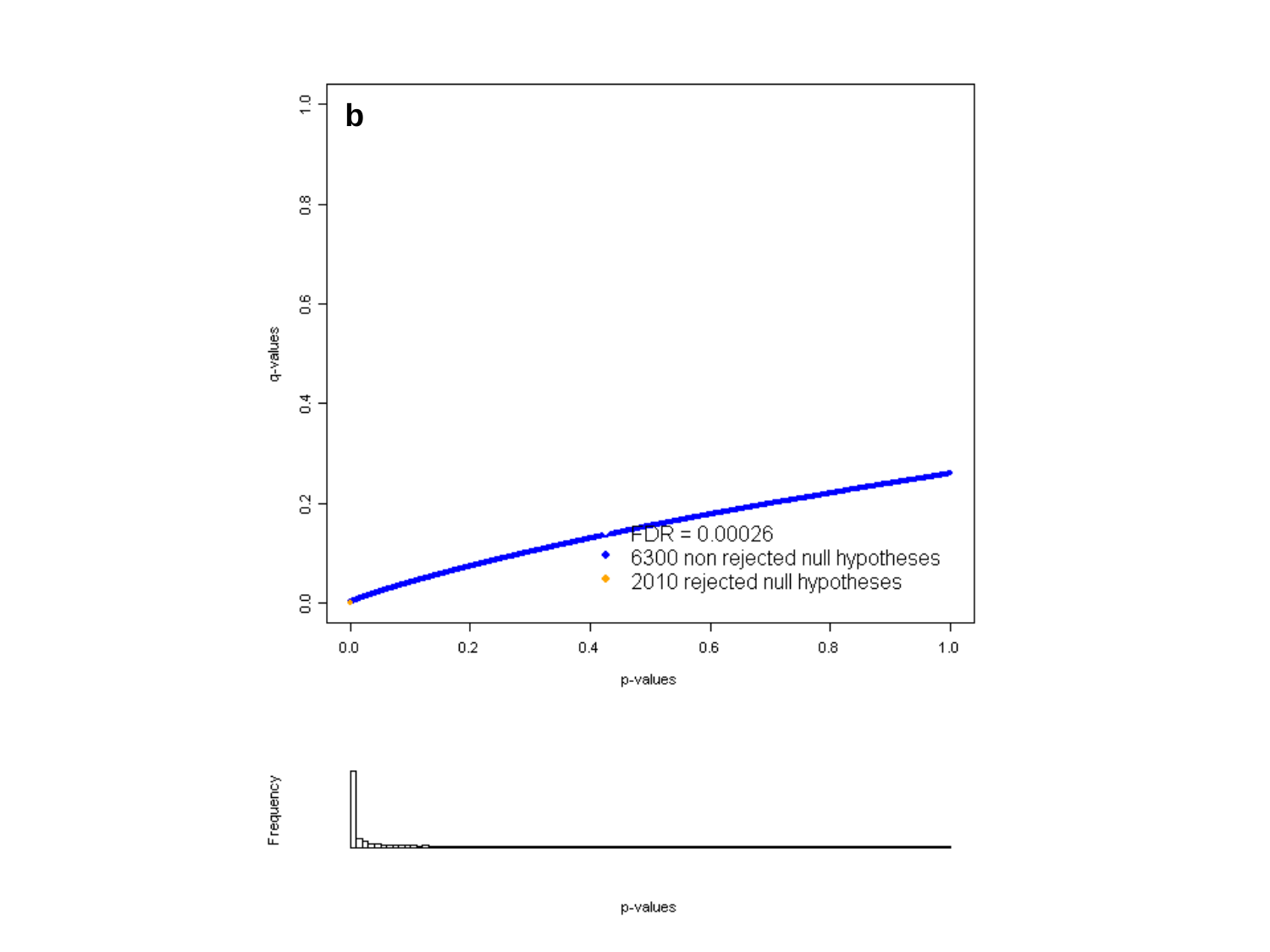

b

## Slide 3
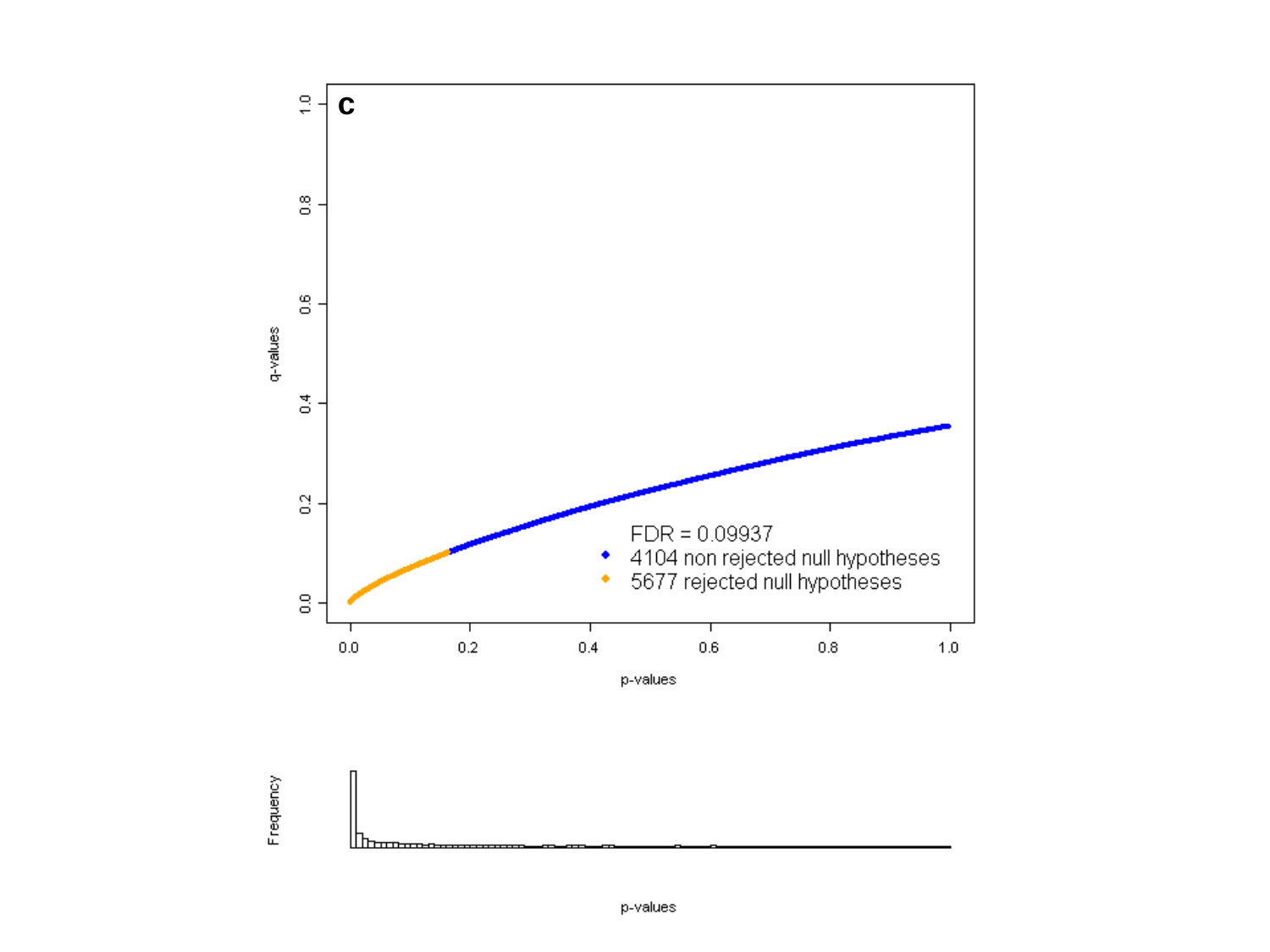

c

Supplement: Additional file 6 — False Discovery Rate (FDR) estimation. The plot shows the histogram of the p-values derived from the Limma moderated t statistics, q-values versus p-values and the expected proportion of FDR estimated on Affymetrix (a), Illumina (b) and RNG-86 (c) platforms. The proportion of true null hypotheses (∏0) was estimated to be 0.273, 0.259 and 0.355 on the Affymetrix, Illumina and RNG-86, respectively. [file 1471-2164-9-302-S6.ppt]

## Slide 1
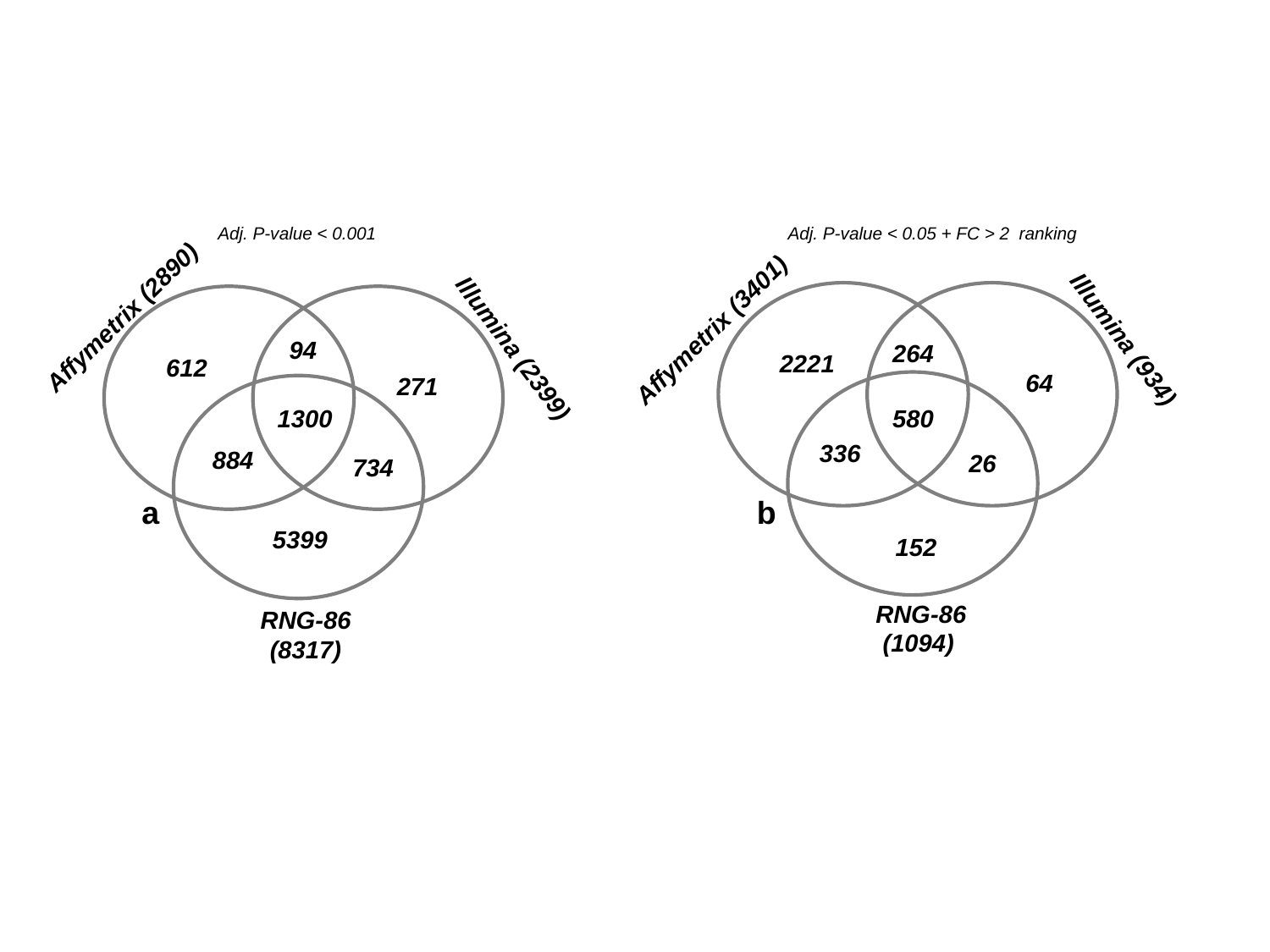

Affymetrix (2890)
 94
Illumina (2399)
612
271
 1300
884
734
5399
RNG-86 (8317)
Illumina (934)
 264
 2221
64
 580
 336
26
152
RNG-86
 (1094)
Affymetrix (3401)
Adj. P-value < 0.001
Adj. P-value < 0.05 + FC > 2 ranking
a
b

Supplement: Additional file 8 — Degree of overlap in lists of differentially expressed among the three platforms. Venn diagrams of the number of genes identified as differentially expressed between monocyte and macrophage samples. Analysis was performed on all probes represented on each platform Affymetrix: 54,613 probe sets, Illumina: 47,296 probe IDs, RNG/MRC: 25,951 (only control probes and bad spots were filtered out). Results are shown for adjusted P-value < 0.001 threshold (a) and adjusted P-value < 0.05 combined to fold change > 2 (b). [file 1471-2164-9-302-S8.ppt]
