# Supplementary material for: Performance comparison of two microarray platforms to assess differential gene expression in human monocyte and macrophage cells
Source: BMC Genomics. 2008 Jun 25;9:302. doi: 10.1186/1471-2164-9-302 (PMC2464609; doi:10.1186/1471-2164-9-302)
Supplement: Additional file 9 — Gene Ontology enrichment comparison. GO enrichment comparison of the lists of differentially expressed genes selected using two criteria. [file 1471-2164-9-302-S9.doc]

Table 4 (a). Gene Ontology (GO) comparison of lists of genes selected using the Best3800 criterion for each platform – Analyses were performed using the common list of transcripts.

| **GO Biological Processes** | **Affymetrix Ref list (18373)** | **Affy list**  **(3735)*** | **Affy P-value** | **Illumina Ref list (16592)** | **Illum list (3756)** | **Illum P-value** | **RNG Ref list**  **(17550)** | **RNG-86 list**  **(3549)** | **RNG-86 P-value** |
| --- | --- | --- | --- | --- | --- | --- | --- | --- | --- |
| Immunity and defense | 1154 | 318 | 1.51E-06 | 1124 | 351 | 4.69E-08 | 1123 | 326 | 2.88E-09 |
| Protein metabolism and modification | 2426 | 584 | 0.000307 | 2316 | 624 | 7.82E-05 | 2340 | 600 | 2.51E-08 |
| Intracellular protein traffic | 874 | 231 | 0.00148 | 823 | 233 | 0.0121 | 846 | 239 | 8.42E-06 |
| Carbohydrate metabolism | 520 | 132 | (ns) | 502 | 147 | 0.04 | 496 | 143 | 0.000828 |
| Electron transport | 221 | 50 | (ns) | 217 | 69 | (ns) | 212 | 72 | 0.000836 |
| Apoptosis | 470 | 130 | 0.0122 | 457 | 127 | (ns) | 458 | 132 | 0.00168 |
| Oxidative phosphorylation | 63 | 13 | (ns) | 62 | 28 | (ns) | 61 | 30 | 0.00205 |
| Lipid, fatty acid and steroid metabolism | 673 | 189 | 0.000292 | 647 | 196 | 0.00121 | 655 | 178 | 0.00215 |
| Protein folding | 141 | 39 | (ns) | 141 | 46 | (ns) | 139 | 53 | 0.00242 |
| Coenzyme and prosthetic group metabolism | 146 | 50 | 0.0121 | 145 | 45 | (ns) | 143 | 51 | 0.00374 |
| Other metabolism | 496 | 141 | 0.00225 | 478 | 142 | 0.0282 | 483 | 134 | 0.00719 |
| Protein biosynthesis | 308 | 58 | (ns) | 293 | 84 | (ns) | 284 | 88 | 0.0136 |
| Pre-mRNA processing | 246 | 49 | (ns) | 235 | 71 | (ns) | 230 | 74 | 0.0159 |
| Intracellular signaling cascade | 818 | 217 | 0.00949 | 779 | 214 | (ns) | 802 | 208 | 0.0326 |
| Protein modification | 1046 | 272 | 0.0045 | 985 | 285 | 0.00308 | 1016 | 255 | 0.0462 |
| Cell cycle | 836 | 212 | 0.0246 | 791 | 210 | (ns) | 803 | 187 | (ns) |

For each platform, the list of differentially expressed genes was statistically compared to the list of all genes represented on the array. The table is ordered by the adjusted P-value of the test of association between RNG-86 list and GO classes. Sixteen GO classes were significantly (Pc < 0.05) enriched in at least one of the three lists; 10, 7 and 15 of these processes were enriched in the Affymetrix, Illumina and RNG-86 lists, respectively.

(*) Some genes (unmapped gene IDs) were excluded by the Panther system from the functional analysis; this can explain the difference observed in the number of genes reported in GO comparison tables and the numbers of genes reported in **Table 1**.

Table 4 (b). Gene Ontology (GO) comparison of lists of genes selected using the p<0.001 criterion for each platform – Analyses were performed using the common list of genes.

| **GO Biological Processes** | **Affymetrix  Ref List (18373)** | **Affy list (1876)** | **Affy P.value** | **Illumina Ref List (16592)** | **Illum list (1990)** | **Illum P.value** | **RNG Ref List (17550)** | **RNG-86 list (5198)** | **RNG-86 P.value** |
| --- | --- | --- | --- | --- | --- | --- | --- | --- | --- |
| Intracellular protein traffic | 874 | 103 | (ns) | 823 | 119 | (ns) | 846 | 349 | 2.84E-08 |
| Protein metabolism and modification | 2426 | 283 | (ns) | 2316 | 342 | 0.00094 | 2340 | 836 | 2.32E-07 |
| Signal transduction | 2973 | 374 | 0.00031 | 2866 | 350 | (ns) | 2979 | 733 | 2.83E-07 |
| Pre-mRNA processing | 246 | 14 | (ns) | 235 | 25 | (ns) | 230 | 115 | 1.67E-05 |
| mRNA splicing | 179 | 7 | (ns) | 170 | 20 | (ns) | 168 | 87 | 0.000193 |
| Oxidative phosphorylation | 63 | 3 | (ns) | 62 | 10 | (ns) | 61 | 39 | 0.00182 |
| Protein complex assembly | 61 | 9 | (ns) | 59 | 9 | (ns) | 60 | 38 | 0.00285 |
| Cell adhesion | 538 | 63 | (ns) | 526 | 59 | (ns) | 539 | 115 | 0.00314 |
| Other metabolism | 496 | 67 | (ns) | 478 | 80 | (ns) | 483 | 189 | 0.00348 |
| Electron transport | 221 | 19 | (ns) | 217 | 33 | (ns) | 212 | 93 | 0.00611 |
| Carbohydrate metabolism | 520 | 69 | (ns) | 502 | 75 | (ns) | 496 | 190 | 0.00943 |
| Protein modification | 1046 | 136 | (ns) | 985 | 169 | 0.00045 | 1016 | 364 | 0.0219 |
| Coenzyme and prosthetic group metabolism | 146 | 25 | (ns) | 145 | 20 | (ns) | 143 | 64 | 0.0342 |
| Protein folding | 141 | 17 | (ns) | 141 | 26 | (ns) | 139 | 65 | 0.0499 |
| Cell cycle | 836 | 117 | 0.0154 | 791 | 106 | (ns) | 803 | 283 | (ns) |
| Immunity and defense | 1154 | 189 | 7.16E-09 | 1124 | 205 | 9.28E-08 | 1123 | 383 | (ns) |
| Nucleoside, nucleotide and nucleic acid metabolism | 2806 | 226 | 0.00113 | 2643 | 264 | 0.0157 | 2687 | 859 | (ns) |
| Apoptosis | 470 | 77 | 0.00173 | 457 | 75 | (ns) | 458 | 163 | (ns) |
| Lipid, fatty acid and steroid metabolism | 673 | 107 | 0.00024 | 647 | 119 | 0.00015 | 655 | 223 | (ns) |
| Intracellular signaling cascade | 818 | 118 | 0.0224 | 779 | 118 | (ns) | 802 | 272 | (ns) |
| Oncogenesis | 389 | 64 | 0.0063 | 374 | 59 | (ns) | 376 | 116 | (ns) |

For each platform, the list of differentially expressed genes was statistically compared to the list of all genes represented on the array. The table is ordered by the adjusted P-value of the test of association between RNG-86 list and GO classes. Twenty one GO classes were significantly (Pc < 0.05) enriched in at least one of the three lists; 8, 5 and 14 of these processes were enriched in the Affymetrix, Illumina and RNG-86 lists, respectively.

Table 4 (c). Gene Ontology (GO) comparison of lists of genes selected using the p<0.001 criterion for each platform – Analyses were performed using the whole content of each platform (only control probes and bad spots were filtered out).

| **GO Biological Processes** | **Affymetrix Ref list (18373)** | **Affy list (2802)** | **Affy P.value** | **Illumina Ref list (16592)** | **Illum list 2391** | **Illum P-value** | **RNG-86 Ref List.txt**  **(17550)** | **RNG-86 list (8096)** | **RNG-86 P-value** |
| --- | --- | --- | --- | --- | --- | --- | --- | --- | --- |
| Protein metabolism and modification | 2426 | 435 | 0.00658 | 2316 | 408 | 0.00035 | 2340 | 1219 | 0.000121 |
| Protein biosynthesis | 308 | 38 | (ns) | 293 | 52 | (ns) | 284 | 176 | 0.0133 |
| Immunity and defense | 1154 | 226 | 0.00316 | 1124 | 245 | 4.99E-09 | 1123 | 589 | 0.026 |
| Intracellular protein traffic | 874 | 158 | (ns) | 823 | 153 | 0.0324 | 846 | 452 | 0.0289 |
| Apoptosis | 470 | 108 | 0.00093 | 457 | 84 | (ns) | 458 | 235 | (ns) |
| Cell cycle | 836 | 163 | 0.0341 | 791 | 129 | (ns) | 803 | 397 | (ns) |
| Lipid, fatty acid and steroid metabolism | 673 | 136 | 0.0237 | 647 | 140 | 7.25E-05 | 655 | 310 | (ns) |
| Nucleoside, nucleotide and nucleic acid metabolism | 2806 | 402 | (ns) | 2643 | 327 | 0.0371 | 2687 | 1245 | (ns) |
| Protein modification | 1046 | 197 | (ns) | 985 | 189 | 0.00825 | 1016 | 511 | (ns) |

The table is ordered by the adjusted P-value of the test of association between RNG-86 list and GO classes and only GO classes significantly (Pc < 0.05) enriched in at least one of the three lists are listed.
