# Supplementary material for: Performance comparison of two microarray platforms to assess differential gene expression in human monocyte and macrophage cells
Source: BMC Genomics. 2008 Jun 25;9:302. doi: 10.1186/1471-2164-9-302 (PMC2464609; doi:10.1186/1471-2164-9-302)
Supplement: Additional file 10 — Additional material. mRNA preparation, amplification, hybridization to the 3 array types, image processing, raw data and probes filtering. [file 1471-2164-9-302-S10.doc]

## mRNA preparation and amplification

For the isolation of PMBC from anti-coagulated blood and removal of dead cells, a density gradient centrifugation using Ficoll–Paque (GE Healthcare) gradient was used. PMBC were washed with PBS and the supernatant completely removed. The cell pellet was re-suspended in 80 µl of PBS/BSA buffer per 107 total cells and 10 µl of MACS CD14 (Miltenyi Biotec) were added carefully, mixed and incubated for 15 min at 6°-12°C. The cells were washed by adding 10-20X labelling volume of buffer, centrifuged at 300 g for 10 min. The cells were re-suspended in 500 µl of buffer before magnetic separation as described by the manufacturer.

The human monocytes CD14+ obtained after MACS separation were cultured at a concentration of 1x 10^6^ cells/ml in pure RPMI 1640 medium in P6 Petri dishes. After 1h of incubation at 37°C, non adherent cells were removed and the medium was replaced with RPMI 1640 Glu+ medium supplemented with 100U/ml penicillin, 100 µg/ml streptomycin and 10 % FCS, together with 10 ng/ml of macrophage colony stimulating-factor (M-CSF, SIGMA). Cells were incubated for 6 days at 37°C/5% CO2 to induce the phagocytic differentiation.

## RNA preparation

Total RNA was extracted from monocytes and macrophages using RNeasy mini kit of Qiagen according to the manufacturer’s instructions. All RNA preparations were checked with Agilent Bioanalyser (RNA 6000 nano-kit) and only RNA with RNA Integrity Number (RIN)>8 was been accepted for RNA amplification.

## Amplification of RNA, hybridization, image processing, and raw data extraction for the 3 platforms

**Illumina:** The Illumina TotalPrep RNA Amplification kit (Ambion, UK) was used for all samples using 200 ng of total RNA as starting material. Briefly, the procedure consists of a reverse transcription step using an oligo(dT) primer bearing a T7 promoter and the high yield ArrayScriptTM reverse transcriptase. The cDNA then undergoes second strand synthesis and clean-up to become a template for in vitro transcription with T7 RNA Polymerase and biotin-NTP mix. Labelled cRNA was then cleaned up and 1.5 µg were hybridized to human 6 beadarrays (Illumina, CA, USA) for 16 hours at 55°C. Following hybridization, beadarrays were washed and stained with streptavidin-Cy3 (GE Healthcare, UK). Fluorescent images were obtained with a Beadarray reader and processed with the BeadScan software (Illumina, CA, USA).

**Affymetrix:** The Genechip expression two-cycle target labelling kit (Affymetrix, CA, USA) was used for all samples according to manufacturer’s instructions. Briefly, the procedure consists of two cycles of reverse transcription followed by in vitro transcription (IVT). For each sample, 50 ng of total RNA were reverse transcribed using a T7-oligo (dT) promoter primer in a first strand cDNA synthesis. Following RNase H-mediated second strand cDNA synthesis, the double stranded cDNA was cleaned up. It then acts as a template for IVT using the MEGAscript T7 kit (Ambion, UK) to generate cRNA. The second cycle started with cRNA reverse transcription using random primers, followed by the second strand cDNA synthesis. The resulting double stranded cDNA was then amplified and labelled using a biotin-NTP mix in the second IVT reaction. The labelled cRNA was then cleaned up and fragmented. 15 µg of cRNA were hybridized to Affymetrix Human U133+2.0 array for 16 hours at 45°C. Following hybridization, the arrays were washed and stained with streptavidin-phycoerythrin. Fluorescent images recorded on a GenChip Scanner 3000 and analyzed with the GeneChip Operating software (Affymetrix, CA, USA).

**RNG/MRC:** The Amino allyl message-AmpTMII aRNA Amplification kit (Ambion) was used for all samples. Briefly, the procedure consists of a reverse transcription step using an oligo(dT) primer bearing a T7 promoter and the high yield ArrayScriptTM reverse transcriptase. The cDNA then undergoes second strand synthesis and clean-up to become a template for in vitro transcription with T7 RNA Polymerase. In vitro transcription (IVT) technology was used to generate large amounts of antisens RNA copies of each mRNA sample. 400 ng of all samples were amplified with this procedure according to manufacturers’ instructions. Labelled cRNA was generated with the CyScribe Post-labelling kit (GE Healthcare UK). All samples were labelled with cyanine 5 (Cy5), whereas a reference cRNA was labelled with Cy3. To generate a reference cRNA, 1µg total RNA from 100 (50 monocytes and 50 macrophages) different samples were mixed, then 750ng mixed total RNA was amplified and labelled with cyanine 5 (Cy3) (reactions were carried out and pooled to generate a sufficient stock available for all hybridizations). Hybridizations were performed using the Agilent in situ hybridization kit. For each sample, 1 µg of Cy5-labeled, linearly amplified cRNA was mixed with equal amounts of Cy3-labelled, linearly amplified reference cRNA. The mixture was then fragmented by incubation with the fragmentation buffer at 60°C for 30 minutes. An equal amount of 2x hybridization buffer was added to the fragmented cRNA mixture and hybridized to whole-genome oligo arrays (Human-25K, Réseau National des Génopoles, France) at 62°C for 17 hours. Fluorescent images of hybridized microarrays were obtained with a GenePix 4000 Axon Instruments scanner and analyzed with the GenePix software (Axon Instruments).

**Probe filtering**

Three types of probes filtering were applied according to the type of the platform. For the three platforms, control probes were removed prior to the statistical analysis. The number of probes filtered out and included in the statistical analyses for each platform is reported in Table 3 online **[See Additional file 5]**. For the RNG/MRC array, the GenePix software used in the extraction of raw data, generated flags which are a qualitative indicators of the quality of the spots; ‘bad’, ‘not found’ and ‘absent’ spots were removed from further analysis. For Illumina and Affymetrix we also performed filtering based on qualitative calls; for the Affymetrix expression data, the Bioconductor package PANP (Presence-Absence calls with Negative Probesets) was used to compute detection calls which consists of qualitative measure indicating whether the transcript was present (P), absent (A) or marginal (M). 34565 probe sets corresponding to ~ 63.22 % of all probe sets represented on the Affymetrix array were designated absent in the 10 samples (i.e. their expression levels were below the threshold of detection). For Bead expression data from Illumina, the qualitative metric of detection provided by the array manufacturer consists of a detection score which estimates the probability that a given target sequence is expressed (above the background) in a sample. These scores are computed relative to the distribution of signal of the negative controls. Detection score close to 1 indicates a high confidence of detection above background, in this paper we used a non-stringent threshold of detection; we considered probes having detection score < 0.80 in the 10 samples as not detected. 11857 Illumina probe IDs (~ 25.07% of the total probes on the array) were found to be not expressed in the 10 samples at a detection score <0.80. Filtering on detection call was used to assess the concordance of detection calls within and between platforms and to exclude probes which were called “absent” in the differential analysis performed on a subset of transcripts common to the three platforms.
